# Clinically important change on the Unified Dyskinesia Rating Scale among patients with Parkinson's disease experiencing dyskinesia

**DOI:** 10.3389/fneur.2022.846126

**Published:** 2022-10-20

**Authors:** Rajesh Pahwa, Susan Fox, Robert A. Hauser, Stuart Isaacson, Judy Lytle, Reed Johnson, Lily Llorens, Andrea E. Formella, Caroline M. Tanner

**Affiliations:** ^1^University of Kansas Medical Center, Kansas City, KS, United States; ^2^Toronto Western Hospital, UHN and University of Toronto, Toronto, ON, Canada; ^3^University of South Florida, Tampa, FL, United States; ^4^Parkinson's Disease and Movement Disorders Center, Boca Raton, FL, United States; ^5^Formerly With Adamas Pharmaceuticals, Inc., Emeryville, CA, United States; ^6^Supernus Pharmaceuticals, Inc., Rockville, MD, United States; ^7^University California San Francisco and San Francisco Veterans Affairs Medical Center, San Francisco, CA, United States

**Keywords:** minimal clinically important change, minimal clinically importance difference, Parkinson's disease, dyskinesia, amantadine, Movement Disorders, Unified Dyskinesia Rating Scale, anti-Parkinson's agents

## Abstract

**Background:**

The Unified Dyskinesia Rating Scale (UDysRS) evaluates dyskinesia in patients with Parkinson's disease (PD). A minimal clinically important change (MCIC)—the smallest change in a treatment outcome that a patient considers important—remains undefined for the UDysRS.

**Objective:**

To utilize pivotal amantadine delayed-release/extended-release (DR/ER) trial data to derive MCICs for the UDysRS total score in patients with PD experiencing dyskinesia.

**Methods:**

Pivotal trials included PD patients with ≥1 h daily ON time with troublesome dyskinesia and baseline scores ≥2 on the Movement Disorder Society-Unified Parkinson's Disease Rating Scale (MDS-UPDRS) Part IV, item 4.2. Patients randomized to amantadine DR/ER or placebo completed two consecutive 24-h diaries before each clinic visit and were evaluated during ON time with dyskinesia using the UDysRS, MDS-UPDRS, and Clinician Global Impression of Change (CGI-C). The UDysRS changes from baseline to week 12 were anchored to corresponding changes in MDS-UPDRS item 4.2 scores. A minimal clinically important improvement in the CGI-C and diary-reported ON time with troublesome dyskinesia (≥0.5 h) were supportive anchors. Receiver operating characteristic curves determined the UDysRS change values optimizing sensitivity and specificity to at least minimal improvement on each anchor.

**Results:**

The analyses included 196 patients. Week 12 UDysRS total score reduction of ≥8 points corresponded to at least minimal MDS-UPDRS item 4.2 improvement. UDysRS reduction of ≥9 points corresponded to decreased ON time with troublesome dyskinesia of ≥0.5 h per patient diaries, and UDysRS reduction of ≥10 points corresponded to at least minimal improvement on the CGI-C.

**Conclusion:**

Anchored to the MDS-UPDRS Part IV, item 4.2, an 8-point reduction in the UDysRS total score can be considered an MCIC for PD patients with dyskinesia.

## Introduction

In Parkinson's disease (PD), levodopa remains the most efficacious treatment for motor symptoms, but levodopa-induced dyskinesia (LID) commonly develops with long-term use (1). Dyskinesia can negatively impact physical, psychological, and social health, increase the cost of care, and reduce the quality of life (1–3). Management strategies for dyskinesia include fractionating or lowering levodopa dosage, modifying the dose of other PD medications, surgical interventions (including deep brain stimulation), or adding an anti-dyskinetic glutaminergic antagonist such as the uncompetitive N-methyl-D-aspartate receptor antagonist amantadine (4).

The delayed-release/extended-release (DR/ER) formulation of amantadine (Gocovri^®^ amantadine ER capsules) is the only medication indicated for the treatment of dyskinesia (2017) (5). It is also indicated for adjunctive treatment to carbidopa/levodopa for OFF episodes (2021) and is the only medication currently available that improves OFF episodes without increasing risk for dyskinesia (5). It is designed to be taken once daily at bedtime, with delayed release to minimize effects on sleep, followed by a gradual increase in plasma concentrations overnight with high plasma levels on awakening that are sustained throughout the day when patients are likely to experience motor complications (5, 6).

In the recent pivotal trials leading to the FDA approval of amantadine DR/ER, the primary outcome measure was change from baseline to week 12 in the Unified Dyskinesia Rating Scale (UDysRS) total score (7, 8). The UDysRS is a four-part, reliable, comprehensive, clinimetric rating scale with acceptable inter- and intra-rater consistency designed to evaluate dyskinesia in patients with PD experiencing LID (9). Part I assesses the patient's self-report of ON dyskinesia severity, while Part II evaluates the patient's self-report of OFF dystonia severity over the previous week (9, 10). In Part III, the clinician rates the patient's impairment due to dyskinesia severity over seven body regions, while in Part IV the clinician rates the patient's disability due to dyskinesia severity based on the observation of four activities: talking, drinking from a cup, dressing, and walking (9, 10). All patient-rated (historical) and clinician-assessed (clinical) UDysRS items are scored on a scale of 0–4 (0 = normal, 1 = slight, 2 = mild, 3 = moderate, 4 = severe) (9). Written anchors associated with each item guide patients or clinicians in appropriate response selection (9). The UDysRS total score is calculated by summing the scores of all four parts, with a possible total ranging from 0 to 104 (9, 11).

Statistically significant improvements in efficacy outcomes do not necessarily denote clinical importance (11, 12). To determine how outcome changes may manifest in patients' everyday lives, it is necessary to establish a minimal clinically important change (MCIC)—the smallest change from baseline in a treatment outcome that a patient perceives as important (11, 13). Establishing an MCIC provides a readily interpretable direct estimate of the change in how a patient feels and functions secondary to treatment, which is not always known when study results are presented by comparing mean endpoint changes among treatment groups (14). An MCIC is not only relevant during the clinical assessment of patients but also integral to new drug development, facilitating efficacy interpretation and informing appropriate labeling claims (11, 15).

The EASE LID and EASE LID 3 studies were pivotal phase 3 trials assessing the efficacy and safety of amantadine DR/ER in patients with PD experiencing dyskinesia (7, 8). Both studies used the UDysRS total score as the primary outcome measure and demonstrated a significantly greater mean improvement in the UDysRS total score from baseline to week 12 for amantadine DR/ER vs. placebo (7, 8). Both studies showed amantadine DR/ER treatment reduced both dyskinesia and OFF time and increased ON time without troublesome dyskinesia (7, 8).

Change from baseline in the Movement Disorder Society-Unified Parkinson's Disease Rating Scale (MDS-UPDRS) score was a secondary outcome measure in these trials (7, 8). The MDS-UPDRS comprises four parts in which clinicians and patients evaluate the impact of PD signs and symptoms on patients' activities of daily living and movement (16). Part IV of the MDS-UPDRS assesses motor fluctuations and dyskinesia (16).

While previous research by Makkos et al. (11) and Mestre et al. (14) suggested minimal clinically important differences (MCIDs) for portions of the UDysRS, there is currently no established MCIC for the UDysRS total score. We utilized outcome data from the pooled pivotal amantadine DR/ER trials in patients with LID to identify MCICs for the UDysRS total score, anchored against a minimal change in the MDS-UPDRS Part IV, item 4.2 (Functional Impact of Dyskinesia), and against changes in time spent with dyskinesia from patient diaries and in Clinical Global Impression of Change (CGI-C) scores.

## Materials and methods

### Study design

These studies were conducted according to the ethical principles of the Declaration of Helsinki with approvals from local ethics committees or Institutional Review Boards. All patients provided informed consent. EASE LID (NCT02136914) and EASE LID 3 (NCT02274766) were randomized, double-blinded, placebo-controlled phase 3 trials lasting ≥13 weeks. The pooled trial database included assessment time points common to both trials (weeks 2, 8, and 12) and was used for the MCIC analysis (7, 8). For each trial, week 12 was the pre-specified primary outcome assessment time point. Eligible patients included those who were 30–85 years old with a clinical diagnosis of PD and receiving stable doses of a levodopa preparation administered ≥3 times daily (7, 8). The inclusion criteria also required a patient score ≥2 on Part IV, item 4.2, of the MDS-UPDRS indicating at least mild functional impairment due to dyskinesia and the patient experiencing at least 1 h per day (≥two 30-min periods) of troublesome dyskinesia, recorded between 9:00 am and 4:00 pm, in 24-h patient diaries on 2 consecutive days before study Day 1 (7, 8). Other key inclusion and exclusion criteria have been previously published (7, 8). The pooled modified intent-to-treat population included all randomized patients dosed with amantadine DR/ER or placebo who had ≥1 postbaseline UDysRS assessment.

### Assessments

The UDysRS and MDS-UPDRS were completed at baseline and at each clinic visit. The CGI-C was completed at each clinic visit. Additionally, patients completed home diaries where they recorded their predominant motor state over each 30-min interval for 2 consecutive days (48 total h) at baseline and prior to each follow-up visit (7, 8). Patient training and diary concordance testing were completed at the screening to ensure patient or care partner understanding of each motor state and to confirm agreement among patients and raters (7, 8).

Part IV of the MDS-UPDRS assesses motor complications; item 4.2 specifically evaluates the functional impact of dyskinesia (16). Clinicians were asked to determine the degree to which dyskinesia impacts patient daily function in terms of activities and social interactions using information from the patient and care partner as well as observations during the clinic visit (16). Responses were anchored and scored similarly to the UDysRS questions, such that possible scores reflect a standard progression of disability, from 0 (normal) to 4 (severe impact), where a score of 2 (mild impact) indicates that symptoms are sufficient to cause at least modest functional impairment (16, 17).

Because the EASE LID and EASE LID 3 trials did not include Patient Global Impression of Change (PGI-C) ratings, which are often utilized to anchor MCIC determinations, the MDS-UPDRS Part IV, item 4.2, was selected as the most appropriate anchor in the calculation of MCICs for the UDysRS total score, as it is a measure of functional change due to dyskinesia based on patient impressions over the previous week, and is associated with a meaningful change in disability (16, 18). The CGI-C and diary measures of change in troublesome dyskinesia were chosen as supportive anchors (sensitivity assessment).

### Statistical analysis

Prior to determining the MCIC, Pearson's and Spearman's correlations between the changes from baseline in the UDysRS total score and the changes from baseline in the MDS-UPDRS Part IV, item 4.2, were evaluated to determine the appropriateness of this measure as the primary anchor (11, 18). Descriptive statistics were also obtained for the changes from baseline in the UDysRS at each level of change in the MDS-UPDRS Part IV, item 4.2, as well as in the CGI-C and diary anchors.

An anchor-based approach (18) was used to determine the minimal clinically important change (MCIC) from baseline in the UDysRS total score denoting improvement, using week 12 results from the pooled phase 3 EASE LID and EASE LID 3 studies, where subjects with early study discontinuation had the last observations (changes in anchor and UDysRS total scores) carried forward. All evaluations were completed by treatment group and for all patients combined.

The MCIC for the UDysRS was first obtained using changes in the MDS-UPDRS Part IV, item 4.2, as the primary anchor. The analyses were then repeated using the CGI-C and reductions in daily ON time with troublesome dyskinesia at week 12 obtained from the diary data as supportive anchors. For these evaluations, minimal improvement was defined as a reduction of ≥1 point (e.g., a shift from “moderate” to “mild” impairment) on the MDS-UPDRS Part IV, item 4.2, or a score of at least 1 (meaning “minimally improved”) or greater using the CGI-C as an anchor. Because it was unclear whether 30 min (the minimum measured unit of change) indeed constituted minimally “important” improvement in the PD diary evaluations of ON time with troublesome dyskinesia, calculations of MCIC were done using both ≥30 min and ≥1 h of change per day as anchors.

In order to determine the MCIC, receiver operating characteristic (ROC) curves were constructed, plotting sensitivity vs. 1 minus specificity for each 1-point change in the UDysRS score. For each designated anchor, the MCIC denoting improvement was the UDysRS score change that minimized the objective function: $\sqrt{\left[\text{(1–sensitivity)}{}^{2}\;+\text{(1–specificity)}{}^{2}\right]}$. Sensitivity was defined as the percentage of patients with a UDysRS score change equal to or less (more improved) than the value being tested among patients considered at least minimally improved as measured by the given anchor. Specificity was defined as the percentage of patients with a UDysRS score change greater (less improved) than the value being tested among patients not considered at least minimally improved as measured by the given anchor.

The evaluations to determine the MCIC for the UDysRS denoting improvement were repeated for the changes from baseline in each component of the UDysRS (Parts I, II, III, and IV) as well as for the subtotal of Parts I + II (subjective components) and the subtotal of Parts III + IV (objective components).

As the UDysRS total score is a composite of subjective and objective total scores, an additional evaluation was conducted to obtain the change from baseline for the UDysRS total score that would result in the best agreement between these two sub-scores (where agreement indicates whether both patient- and clinician-rated composites improved or not). This concordance/discordance evaluation was conducted using a similar ROC approach, with the objective function replaced by √ {[1-concordance (all subjects)]^2^ + [discordance (all subjects)]^2^}. This value was then used to provide additional characterizations of the MCIC values obtained using the anchor-based approaches.

Evaluations to determine the MCIC from baseline in the UDysRS total score denoting patient worsening were not conducted, as comparatively few subjects experienced any clinical worsening.

## Results

For the pooled EASE LID and EASE LID 3 studies, the modified intent-to-treat population included a total of 196 patients (Supplementary Figure 1) (19). Patients' baseline demographic information and PD characteristics are provided in Table 1.

**Table 1:** Baseline demographics and Parkinson's disease characteristics.

**Baseline characteristic**	**All participants (*N* = 196)**
Age, years	64.7 (34, 82)
Age <65 years, %	45.4
Male, %	55.6
Age at PD diagnosis, years	55.5 (29, 75)
Duration of levodopa treatment, years	7.7 (1.1, 20.3)
Duration of dyskinesia, years	3.8 (0.1, 14)
UDysRS total score	40.1 (8, 76)
MDS-UPDRS Part IV, item 4.2, score	2.5 (2, 4)
MDS-UPDRS Part IV, item 4.2, score = 2, *n* (%)	97 (49.5)
MDS-UPDRS Part IV, item 4.2, score ≥3, *n* (%)	99 (50.5)
ON time with troublesome dyskinesia, hours	4.9 (0, 13.3)
ON time without troublesome dyskinesia, hours	8.4 (0, 15.3)
OFF time, hours	2.8 (0, 9.5)
Levodopa total daily dose, mg, median (min, max)	612.5 (130, 3100)
Sleep time, hours	8.0 (3.6, 12.8)

Data shown as mean (min, max) unless otherwise indicated.

Max, maximum; min, minimum; MDS-UPDRS, Movement Disorder Society-Unified Parkinson's Disease Rating Scale; PD, Parkinson's disease; UDysRS, Unified Dyskinesia Rating Scale.

### Correlations between changes in UDysRS and anchors

**Figure 1:**
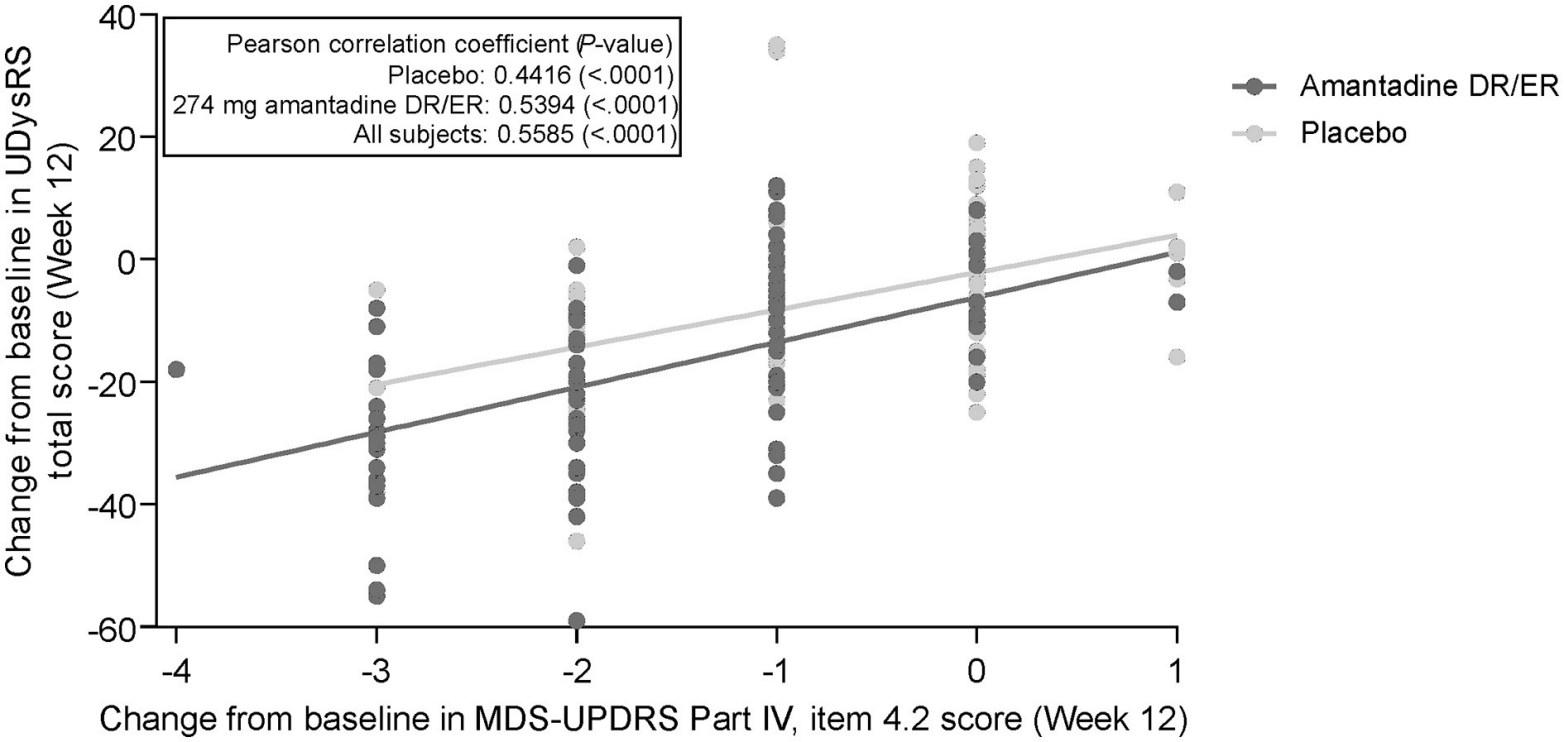
Correlation of change from baseline in UDysRS total score and MDS-UPDRS Part IV, item 4.2. 274 mg amantadine base is approximately 340 mg amantadine hydrochloride. DR, delayed-release; ER, extended-release; MDS-UPDRS, Movement Disorder Society-Unified Parkinson's Disease Rating Scale; UDysRS, Unified Dyskinesia Rating Scale.

Individual changes in the UDysRS total score from baseline to week 12 are positively correlated with changes in the MDS-UPDRS Part IV, item 4.2, ratings (Figure 1). Pearson's correlation coefficient value was 0.44 (*P* < 0.0001) for the placebo group, 0.54 (*P* < 0.0001) for the amantadine DR/ER group, and 0.56 (*P* < 0.0001) for all patients. Mean changes from baseline in the UDysRS are summarized in Table 2 for each incremental level of improvement in the MDS-UPDRS Part IV, item 4.2, at week 12. The positive correlation shown in Figure 1 is also apparent in Table 2, as larger mean decreases in the UDysRS were seen with larger decreases (i.e., improvements) in the MDS-UPDRS Part IV, item 4.2. Generally, more subjects in the amantadine DR/ER group demonstrated improvements as compared to the placebo group. Improved subjects in the amantadine DR/ER group also had larger mean changes in the UDysRS total score than in the placebo group at each level of improvement for the MDS-UPDRS Part IV, item 4.2, anchor.

**Table 2:** Mean changes in the UDysRS total score for each level of change in the MDS-UPDRS Part IV, item 4.2.

**Treatment group**	**Change in MDS-UPDRS Part IV, item 4.2 at week 12**	***n***	**Change in UDysRS total score at week 12, mean (SD)**
**All patients**	−4	1	−18.0 (N/A)
	−3	25	−28.8 (12.7)
	−2	45	−19.4 (12.9)
	−1	63	−8.9 (13.4)
	0	54	−3.5 (10.7)
	1	8	−2.0 (7.7)
**Amantadine DR/ER**	−4	1	−18.0 (N/A)
	−3	21	−29.9 (12.5)
	−2	26	−22.5 (13.2)
	−1	35	−10.5 (12.9)
	0	15	−8.6 (8.5)
	1	2	−4.5 (3.5)
**Placebo**	−3	4	−23.5 (14.2)
	−2	19	−15.1 (11.5)
	−1	28	−7.0 (13.9)
	0	39	−1.6 (10.8)
	1	6	−1.2 (8.8)

Score changes are from baseline to week 12 (using LOCF). DR, delayed-release; ER, extended-release; LOCF, last observation carried forward; MDS-UPDRS, Movement Disorder Society-Unified Parkinson's Disease Rating Scale; N/A, not applicable; SD, standard deviation; UDysRS, Unified Dyskinesia Rating Scale.

Mean changes from baseline in the UDysRS are also summarized in Tables 3, 4 for 30- and 60-min decreases in ON time with troublesome dyskinesia as well as incremental changes in the CGI-C, respectively. Positive correlations are also noted with these supportive anchors, as larger mean decreases from baseline corresponding to improvements in the UDysRS were seen with larger improvements in these anchors. As observed with the MDS-UPDRS Part IV, item 4.2, greater improvements in the UDysRS were seen among patients receiving amantadine DR/ER as compared to the placebo group across the different time points of the ON time with troublesome dyskinesia anchor.

**Table 3:** Mean changes in the UDysRS total score for each threshold of reduction in ON time with troublesome dyskinesia.

**Treatment group**	**Decrease in ON time with troublesome dyskinesia at week 12**	***n***	**Change in UDysRS total score at week 12, mean (SD)**
**All patients** ^**a**^	≥1 h	131	−16.2 (14.0)
	<1 h	59	−3.4 (13.3)
	≥0.5 h	147	−14.9 (14.1)
	<0.5 h	43	−3.1 (14.2)
**Amantadine DR/ER** ^**b**^	≥1 h	77	−19.8 (14.2)
	<1 h	18	−7.5 (13.1)
	≥0.5 h	82	−19.1 (14.3)
	<0.5 h	13	−7.6 (14.5)
**Placebo** ^**c**^	≥1 h	54	−11.0 (11.8)
	<1 h	41	−1.6 (13.1)
	≥0.5 h	65	−9.6 (12.0)
	<0.5 h	30	−1.2 (13.9)

Score changes are from baseline to week 12 (using LOCF). Number of participants with no postbaseline diary: ^a^n = 6, ^b^n = 5, ^c^n = 1.

DR, delayed-release; ER, extended-release; LOCF, last observation carried forward; SD, standard deviation; UDysRS, Unified Dyskinesia Rating Scale.

**Table 4:** Mean changes in the UDysRS total score for each category of CGI-C rating.

**Treatment group**	**CGI-C rating at week 12**	***n***	**Change in UDysRS total score at week 12, mean (SD)**
**All patients**	−3 (marked worsening)	1	−29.0 (N/A)
	−2 (moderate worsening)	5	−15.6 (18.9)
	−1 (minimal worsening)	21	−6.5 (15.6)
	0 (no change)	57	−3.7 (11.5)
	1 (minimal improvement)	40	−10.6 (10.5)
	2 (moderate improvement)	42	−17.0 (12.1)
	3 (marked improvement)	30	−26.4 (16.0)
**Amantadine DR/ER**	−3 (marked worsening)	1	−29.0 (N/A)
	−2 (moderate worsening)	2	−23.0 (19.8)
	−1 (minimal worsening)	4	−12.0 (15.3)
	0 (no change)	17	−8.2 (9.5)
	1 (minimal improvement)	19	−10.4 (11.4)
	2 (moderate improvement)	29	−18.7 (12.5)
	3 (marked improvement)	28	−26.2 (16.4)
**Placebo**	−2 (moderate worsening)	3	−10.7 (20.7)
	−1 (minimal worsening)	17	−5.2 (15.9)
	0 (no change)	40	−1.8 (11.8)
	1 (minimal improvement)	21	−10.8 (9.9)
	2 (moderate improvement)	13	−13.0 (10.6)
	3 (marked improvement)	2	−29.5 (12.0)

Score changes are from baseline to week 12 (using LOCF). CGI-C, Clinical Global Impression of Change; LOCF, last observation carried forward; N/A, not applicable; SD, standard deviation; UDysRS, Unified Dyskinesia Rating Scale.

### MCIC for UDysRS

**Figure 2:**
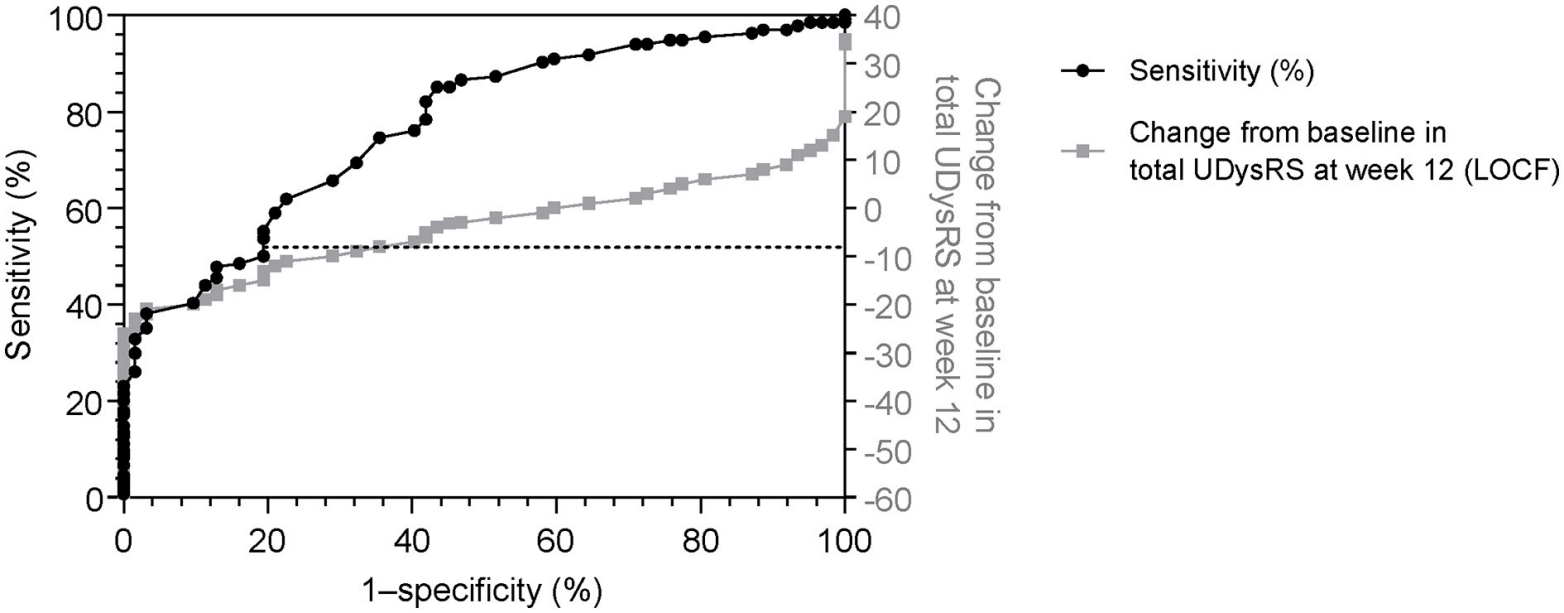
Receiver operating characteristic curve (sensitivity vs. 1–specificity) for change in the UDysRS total score, using the Movement Disorder Society-Unified Parkinson's Disease Rating Scale Part IV, item 4.2 (≥1, minimal) as anchor [black line]. Actual change in UDysRS vs 1–specificity values [gray line] are also shown. 1–specificity values are shown as per the standard for ROC curves. LOCF, last observation carried forward; ROC, receiver operating characteristic; UDysRS, Unified Dyskinesia Rating Scale.

A change of −8 in the UDysRS total score was associated with at least minimal improvement in the MDS-UPDRS Part IV, item 4.2 (sensitivity = 75%, specificity = 65%; Figure 2). An analysis of each component part of the UDysRS yielded MCIC values of −6 for UDysRS Part I, −1 for Part II, −3 for Part III, and −2 for Part IV (Table 5).

**Table 5:** Determination of minimal clinically important change^*^ in the Unified Dyskinesia Rating Scale scores anchored to minimal improvement in the Movement Disorder Society-Unified Parkinson's Disease Rating Scale Part IV, item 4.2, by treatment group and for overall patients.

	**MDS-UPDRS Part IV, item 4.2, score** ≤ **–1**		
	**Sensitivity**	**Specificity**	**MCIC**
**Total UDysRS**			
Placebo	41/51 (80.4%)	31/45 (68.9%)	−5
Amantadine DR/ER	54/83 (65.1%)	13/17 (76.5%)	−12
Total	100/134 (74.6%)	40/62 (64.5%)	−8^†^
**Parts I** **+** **II**			
Placebo	34/51 (66.7%)	29/45 (64.4%)	−3
Amantadine DR/ER	47/83 (56.6%)	13/17 (76.5%)	−9
Total	88/134 (65.7%)	42/62 (67.7%)	−6
**Parts III** **+** **IV**			
Placebo	31/51 (60.8%)	31/45 (68.9%)	−3
Amantadine DR/ER	58/83 (69.9%)	10/17 (58.8%)	−4
Total	94/134 (70.1%)	40/62 (64.5%)	−3
**Part I**			
Placebo	37/51 (72.5%)	27/45 (60.0%)	−2
Amantadine DR/ER	51/83 (61.4%)	12/17 (70.6%)	−6
Total	78/134 (58.2%)	48/62 (77.4%)	−6
**Part II**			
Placebo	24/51 (47.1%)	25/45 (55.6%)	−1
Amantadine DR/ER	41/83 (49.4%)	10/17 (58.8%)	−1
Total	65/134 (48.5%)	35/62 (56.5%)	−1
**Part III**			
Placebo	28/51 (54.9%)	37/45 (82.2%)	−3
Amantadine DR/ER	49/83 (59.0%)	12/17 (70.6%)	−4
Total	85/134 (63.4%)	47/62 (75.8%)	−3
**Part IV**			
Placebo	36/51 (70.6%)	26/45 (57.8%)	−1
Amantadine DR/ER	55/83 (66.3%)	11/17 (64.7%)	−2
Total	78/134 (58.2%)	44/62 (71.0%)	−2

^*^The column labeled “MCIC” shows the threshold of UDysRS change that best predicted minimal improvement in MDS-UPDRS Part IV, item 4.2.

^†^Selected as the MCIC value for minimal improvement in the UDysRS total score.

DR, delayed-release; ER, extended-release; MCIC, minimal clinically important change; MDS-UPDRS, Movement Disorder Society-Unified Parkinson's Disease Rating Scale; UDysRS, Unified Dyskinesia Rating Scale.

**Figure 3:**
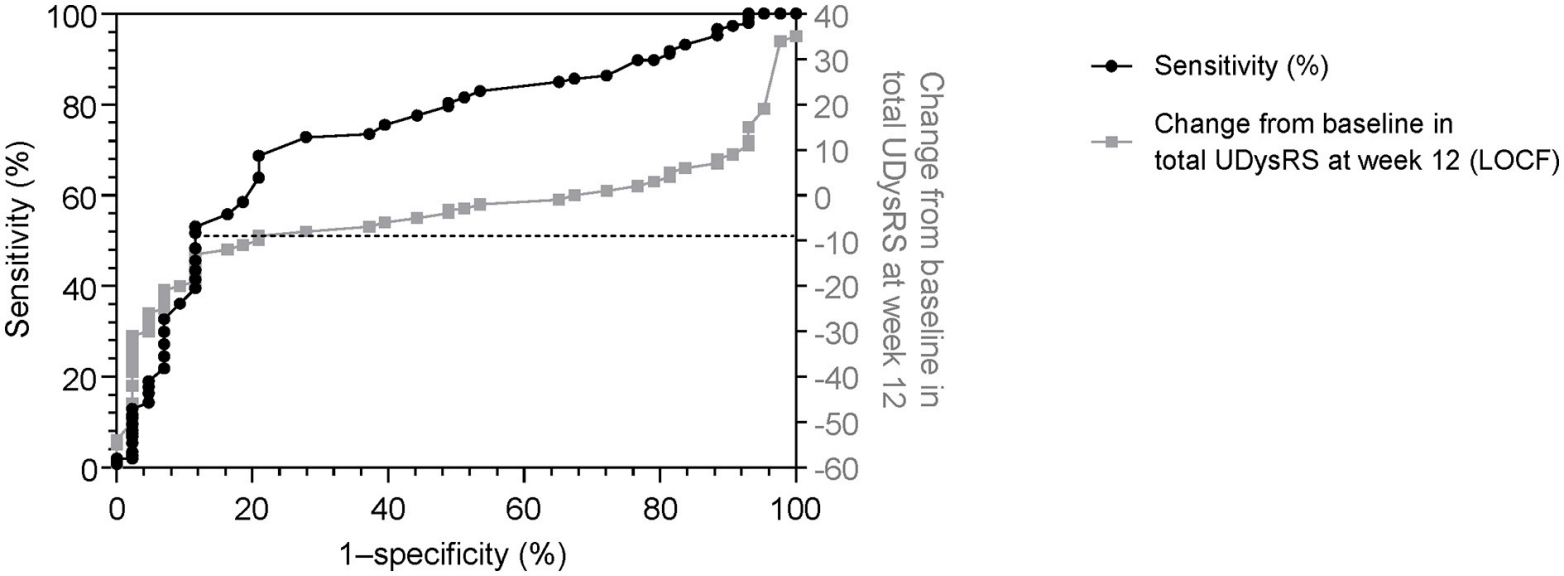
Receiver operating characteristic curve (sensitivity vs. 1–specificity) for change in the UDysRS total score, using patient diary recording of ON time with troublesome dyskinesia (≥0.5-h decrease) as anchor [black line]. Actual change in UDysRS vs 1–specificity values [gray line] are also shown. 1–specificity values are shown as per the standard for ROC curves. LOCF, last observation carried forward; ROC, receiver operating characteristic; UDysRS, Unified Dyskinesia Rating Scale.

Changes of −9 and −10 in the UDysRS total score from baseline to week 12 were associated with minimal decreases of at least 30 min (sensitivity = 69%, specificity = 79%; Figure 3) and 60 min (sensitivity = 69%, specificity = 78%) in ON time with troublesome dyskinesia as measured by patient diaries. A change in the UDysRS total score of −10 was associated with minimal improvement in the CGI-C (sensitivity = 71%, specificity = 68%).

Evaluations of the concordance between the changes from baseline in scores for the UDysRS subjective and objective parts showed that a change of −9 in the UDysRS total score provided the best concordance (77% of subjects) between these two sub-scores.

**Figure 4:**
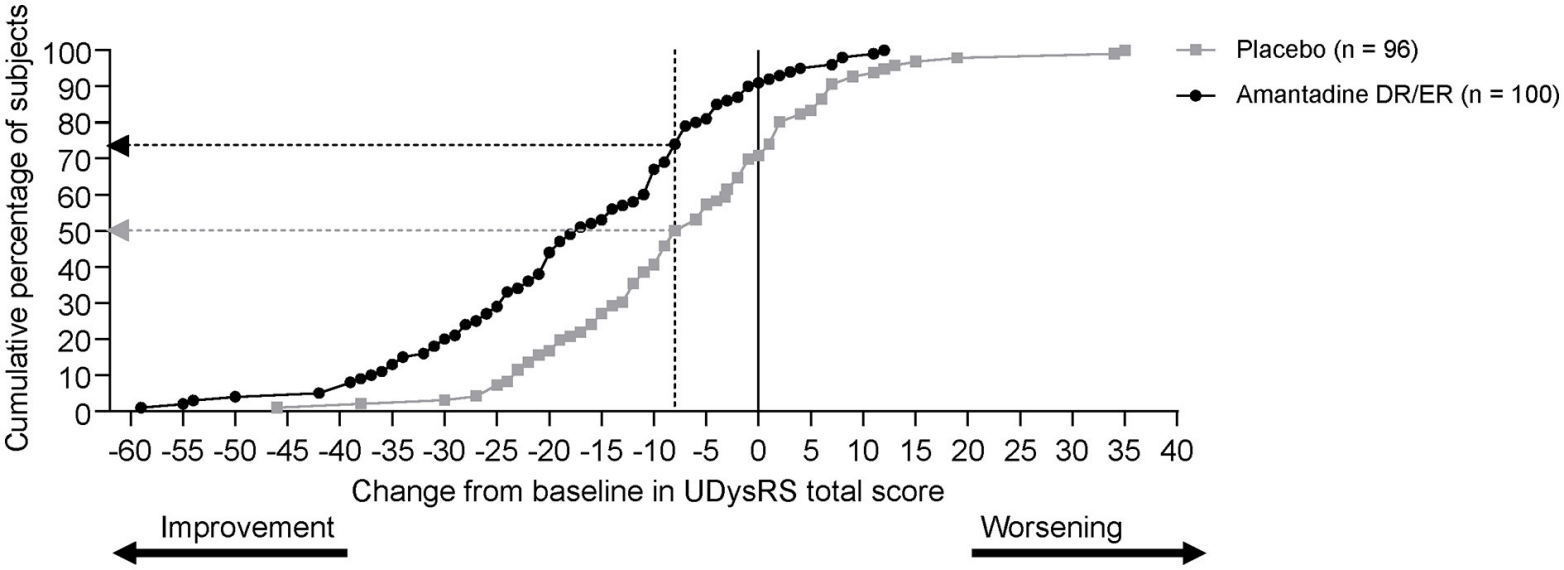
Cumulative distribution of response (UDysRS reduction of at least 8 points) at week 12 (measured using last observation carried forward). DR, delayed-release; ER, extended-release; UDysRS, Unified Dyskinesia Rating Scale.

In total, 74% of patients receiving amantadine DR/ER vs. 50% of patients receiving placebo met the calculated MCIC of −8 for the UDysRS anchored to the MDS-UPDRS Part IV, item 4.2 (Figure 4). The percentage of subjects with decreases from baseline ≥8 points was 62%, and the sensitivity and specificity values for the MCIC of −8 for the UDysRS anchored to the MDS-UPDRS Part IV, item 4.2, yielded positive and negative predictive values of 82% and 54%, respectively.

## Discussion

To our knowledge, this is the first study to derive MCIC values for the UDysRS total score. Calculations based on three different anchors measuring an observable change in patient-perceived function relative to dyskinesia (MDS-UPDRS Part IV, item 4.2; ON time with troublesome dyskinesia; and CGI-C) all returned similar MCIC values for improvement in the UDysRS total score of at least 8–10 points. Furthermore, this level of change provided good concordance between results for the objective and subjective components of the UDysRS.

As dyskinesia can be perceived differently by patients vs. clinicians, the similarity of MCIC calculations derived using these different patient-reported and clinician-reported measures is reassuring (14). Establishing an MCIC helps translate the relevance of changes in clinical trial outcomes in a way that aids clinicians in understanding the importance of treatment interventions (14). These MCIC values are also useful in research to help determine appropriate sample sizes for future clinical trials (20).

A decrease of ≥8 points in the UDysRS total score distinguished an MCIC consistent with relevant improvement in the extent to which dyskinesia impacts patients' daily function, as evaluated by the MDS-UPDRS Part IV, item 4.2. The 62% of subjects with score decreases of ≥8 points and the sensitivity and specificity values obtained using the MDS-UPDRS Part IV, item 4.2, as anchor yielded a positive predictive value (PPV) and a negative predictive value (NPV) of 82% and 54%, respectively, for the MCIC denoting improvement of −8 points. The comparatively greater PPV than NPV is most likely related to the study eligibility criteria (MDS-UPDRS Part IV, item 4.2, ≥2 for study inclusion) and the efficacy objective of the trials.

The MCIC as based on ROC analysis using the MDS-UPDRS Part IV, item 4.2, ratings as an anchor was supported by similar MCIC analyses anchored using the secondary anchors of patient diary self-reports of measurable reduction in time spent with troublesome dyskinesia, as well as CGI-C ratings. The diary analysis found that decreases of ≥9 points and ≥10 points in the UDysRS total score best discriminated patients with and without a 30-min or larger reduction or 60-min or larger reduction in ON time with troublesome dyskinesia, respectively (Supplementary Table 1). Similarly, the CGI-C analysis showed a decrease of ≥10 points in the UDysRS total score best discriminated patients with and without minimal improvement in overall PD signs, including dyskinesia (Supplementary Table 2).

Anchors for MCIC determinations should be independent and clinically relevant tools that can be individually interpreted and correlate with the outcome of interest (11). The MDS-UPDRS Part IV, item 4.2, was used as the primary anchor in the calculation of MCICs for the UDysRS total score because this anchor specifically assesses meaningful improvement in function as perceived by the patient and/or care partner (10, 16). It has been recommended that correlation for a good anchor exceed 0.3 (18). The strong correlation (*r* = 0.56) between changes in the MDS-UPDRS Part IV, item 4.2, and UDysRS scores further supports the choice of item 4.2 as the primary anchor measure. As patients were required to have item 4.2 scores ≥2 at baseline, the opportunity existed for worsening or improvement in this measure (7, 8).

The ROC analysis using the CGI-C returned a similar MCIC for the UDysRS as that of MDS-UPDRS Part IV, item 4.2 (−10 and −8, respectively); however, using CGI-C as the anchor did not show discriminatory power, as the same MCIC (−10) was obtained for most levels of improvement in the CGI-C, as shown in Supplementary Table 2. CGI-C was thus deemed a suboptimal anchor in MCIC determination for the UDysRS as compared to the MDS-UPDRS Part IV, item 4.2. The inability to establish an MCIC that reliably distinguishes various thresholds of change in the CGI-C may be due to the fact that, for this trial, CGI-C assessments evaluated global change in PD, rather than change in dyskinesia specifically (21). Similarly, the use of patient diary-reported reduction in troublesome dyskinesia as a primary anchor for MCIC determination was also determined to be suboptimal, as diaries measure the time spent in each motor state but do not evaluate the patient's perception of these states. Nevertheless, the fact that MCIC calculations using each anchor returned similar values adds a measure of confidence to our MCIC determination.

Previous studies have suggested minimal clinically important differences (MCIDs) for components of the UDysRS. Mestre et al. (14) determined an MCID for Part III of the UDysRS using levodopa infusions to assess patients with dyskinesia. They determined a 2.76-point increase or a 2.32-point decrease represented MCIDs for dyskinesia onset and remission, respectively (14). Our study found that an MCIC for Part III of the UDysRS distinguishing at least minimal improvement in the MDS-UPDRS Part IV, item 4.2, is −3 points, consistent with previous findings (14). Makkos et al. (11) evaluated patients with PD and motor complications every 6 months to suggest MCIDs for the historical (patient-rated) sections of the UDysRS (Parts I and II). They anchored UDysRS change to PGI and determined an MCIC for improvement of −2.5 points and for worsening of +1.5 points for UDysRS Part I, and an MCIC for improvement of −1.5 points and for worsening of +1.5 points for UDysRS Part II (11). The results of our study are partly consistent with those of Makkos et al. (11), calculating an MCIC for improvement of −1 point for UDysRS Part II; however, our MCIC for improvement in UDysRS Part I of −6 points is greater than their threshold. The reasons for this dissimilarity are speculative but could be influenced by differences in baseline severity of the samples, or the fact that patient improvements seen with amantadine DR/ER treatment were relatively large in magnitude, possibly influencing the MCIC calculations. It is important to consider that MCICs are not to be interpreted as definitive values determining whether or not an individual patient is experiencing a meaningful response, but instead as a level of change that best distinguished groups of individuals who either did or did not meet a particular threshold of improvement on the defined anchor.

The MCIC values are derived measures that will vary among studies because they can be determined in multiple ways, such as by calculating the mean score change in patients reporting improvement (called “mean change score”) or by using ROC curves to define the value that best distinguishes patients reporting vs. not reporting improvement (13, 20). The MCIC values may also change depending on the patient population and study circumstances used in their calculation (13, 20). For the pooled EASE LID and EASE LID 3 datasets, the low numbers of patients at the highest and lowest dyskinesia score extremes may have affected the precision of MCIC measurement. For example, all patients were required to have scores ≥2 on the MDS-UPDRS Part IV, item 4.2, at baseline, which meant that fewer patients had the capacity for decline on this anchor. Additionally, the low number of patients reporting worsening of dyskinesia made it impossible to estimate an MCIC for the UDysRS for patient decline. Findings from our analysis should therefore be replicated using other datasets.

It is also of note that, when calculated by treatment group, the MCIC values for patients receiving amantadine DR/ER were consistently higher than those for patients receiving placebo. This is speculated to result from the larger treatment effect observed with amantadine DR/ER as compared to placebo. Highly effective interventions that produce substantial improvements in patient symptoms could yield greater MCIC values, as patients may improve to such a degree that there is a reduced ability to pinpoint a minimally important change. This highlights the challenges of identifying an MCIC using data from subjects receiving highly efficacious therapies. Finally, the pooled studies did not include a Patient Global Impression nor a Health-Related Quality of Life scale. It would be useful to replicate MCIC calculations based on these established anchors.

Furthermore, although our analysis may have benefitted from a larger sample size, it used data from rigorously conducted phase 3 trials with investigators who were trained and experienced in scale administration. The concordance analysis conducted also supports the MCIC values from the primary ROC analysis based on sensitivity/specificity evaluations. Together, these results bode well for the overall MCIC determination. Nevertheless, replication of this work with additional datasets would lend greater certainty to the MCIC values obtained.

In summary, this analysis determined an MCIC for the UDysRS scale using data from well-controlled phase 3 clinical trials assessing a dyskinesia treatment for patients with PD. Evaluation against a minimally relevant change using three different anchors (≥1-point reduction in the MDS-UPDRS Part IV, item 4.2; ≥30- and ≥60-min reductions in patient diary-reported ON time with troublesome dyskinesia; and a rating of at least “minimally improved” on the CGI-C scale) yielded the calculation of similar MCIC values for improvement in the UDysRS total score (8, 9, and 10 points, respectively). Based on these analyses, a decrease of 8 or more points in the UDysRS score represents a minimum threshold for meaningful change in the patient-perceived impact of dyskinesia.

## Data availability statement

The raw data supporting the conclusions of this article will be made available by the authors, without undue reservation.

## Ethics statement

The protocol and study procedures were approved by the following Institutional Review Boards (IRB) and Ethical Committees: Baylor College of Medicine IRB, Beth Israel Medical Center IRB, Biomedical Research Alliance of New York LLC IRB, CEIC del Hospital de la Santa Creu I Sant Pau, Chesapeake Institutional Review Board, Cleveland Clinic IRB, Comite de Protection des Personnes Sud Ouest et Outre Mer ll, Copernicus Group IRB, Ethics Committee of the Innsbruck Medical University, Ethik-Kommission des Fachbereichs Medizin der Phillips-Universtat Marburg, Henry Ford Health System IRB, Johns Hopkins University IRB, Mayo Clinic IRB, Park Nicollet Institute IRB, Penn State College of Medicine IRB, Rush University Medical Center IRB, St. Joseph's Hospital and Medical Center IRB for Human Research, UC Davis Medical Center IRB, University Health Network Ethics Research Board, University of Kansas Medical Center Human Subjects Committee, University of Miami, Human Subjects Research Office, University of Pennsylvania IRB, University of Saskatchewan Biomedical Research Ethics Board, UT Southwestern IRB, Washington University School of Medicine IRB, and Western IRB. The patients/participants provided their written informed consent to participate in this study.

## Author contributions

RP, SF, RH, SI, AF, JL, RJ, LL, and CT wrote sections of the manuscript. AF, JL, RJ, and LL contributed to conception and study design. LL performed the statistical analysis. All authors contributed to manuscript revision, read, and approved the submitted version.

## Funding

This study was funded by Adamas Pharmaceuticals, Inc., Emeryville, CA, United States. Medical writing and editorial support were funded by Supernus Pharmaceuticals, Inc., Rockville, MD, United States.

## Conflict of interest

Author RP reports consulting fees from AbbVie, ACADIA Pharmaceuticals, Acorda Therapeutics, Adamas Pharmaceuticals, Cynapsus Therapeutics, Global Kinetics, Ionis Pharmaceuticals, Lundbeck, Neurocrine Biosciences, St. Jude Medical, Teva Neuroscience, UCB, and US WorldMeds; and research grants from Acorda Therapeutics, Adamas Pharmaceuticals, Avid Radiopharmaceuticals, Boston Scientific, Cala Health, Cynapsus Therapeutics, Kyowa, National Parkinson's Foundation, NIH/NINDS, Parkinson's Study Group, Pfizer, and US WorldMeds. Author SF reports consultant fees from Sunovion and Paladian, speaker honoraria from Teva and Zambon, and advisory board fees from Acadia. Author RH reports consulting fees from AbbVie, Academy for Continued Healthcare Learning, Acadia Pharmaceuticals, Acorda Therapeutics, Adamas Pharmaceuticals, Affriris, Alliance for Aging Research, Alphasights, Amneal Pharmaceuticals, ApoPharma, Aptis Partners LLC, Aranca, Axial Biotherapeutics, Axovant Sciences, Bain Capital, Baron Capital, Brittanna Pharmaceuticals, Cadent Therapeutics, Cerespir, Clearview Healthcare Partners, CNS Ratings LLC, Compass Group, DOB Health LLC, Decision Resources Group, Defined Health, Dellaus Consulting, Denali Therapeutics, Enterin, Evercore, Extera Partners, GE Healthcare, Gerson Lehrman Group, Global Kinetics Corporation, Guide Point Global, Health and Wellness Partners, Healthlogix, Heptares Therapeutics, Huron Consulting Group, lmpax Laboratories, Impel Neuropharma, lnhibikase, lntec Pharma Ltd., International Stem Cell Corporation, lntraMed Educational Group, IQVIA, Jazz Pharmaceutics, Kaiser, Kyowa Kirin Pharmaceutical Development, Kashiv Pharma LLC, L.E.K. Consulting, Lundbeck, Lundbeck A/S, MedaCorp, MEDIQ, Medscape, Medtronic, Michael J Fox Foundation, Mitsubishi Tanabe Pharmaceuticals, Movement Disorder Society, Neuro Challenge Foundation for Parkinson's, Neurocea LLC, Neurocrine Biosciences, Neuroderm, Northwestern University, Orbes, Orbes Medical Group, Orion, Parkinson's Foundation, Parkison Study Group, Partner's Healthcare, Penn Technology Partnership, Pennside Partners, Perception OpCo, Precision Effect, Phase Five Communications, Prescott Medical Group, Prilenia Therapeutics LLC, Projects in Knowledge, Regenera Pharma, SAi Med Partners LLC, Schlesinger Associates, Scion Neurostim LLC, Seagrove Partners, Seelos Therapeutics, Slingshot Insights, Sun Pharma, Sunovion Pharmaceuticals, Teva Pharmaceuticals, The Lockwood Group, USWorldMeds, WebMD, and Windrose Consulting Group; speaker fees from Acorda Therapeutics, Adamas Pharmaceuticals, Amneal Pharmaceuticals, Kyowa Kirin, Neurocrine Biosciences and US WorldMeds; research support from AbbVie, Acorda Therapeutics, AstraZeneca, Axovant Sciences, Biogen, Cavion, Centogene, Cerevance, Cerevel, Covance, Enterin, Global Kinetics Corp., Impax Laboratories LLC, Intec Pharma Ltd., Jazz Pharmaceuticals, Neuroderm, Lundbeck, Michael J Fox Foundation for Parkinson's Research, Neuraly, Pharma2B, F. Hoffman-La Roche, Revance Therapeutics, and Sunovion Pharmaceuticals; grant support from Parkinson's Foundation; and has stock option ownership in Axial Biotherapeutics and Inhibikase Therapeutics. Author SI reports honoraria for CME, and consultant fees, research grants, and/or promotional speaker fees on behalf of AbbVie, Acadia Pharmaceuticals, Acorda, Adamas Pharmaceuticals, Addex Therapeutics, Affiris, Alexva, Allergan, Amarantus Bioscience, Amneal, Aptinyx, Axial, Axovant, Benevolent, Biogen, Britannia Pharmaceuticals, Bukwang, Cadent, Cala, Cerecor, Cerevel, Cipla, Eli Lilly, Enterin, GE Healthcare, Global Kinetics, Impax Pharmaceuticals, Impel, Intec Pharma, Ipsen, IR Labs, Jazz, Kyowa, Lundbeck, Merz, Michael J. Fox Foundation, Mitsubishi Tanabe, Neuralys, Neurocrine Biosciences, Neuroderm, Parkinson's Study Group, Pharma2B, Prilenia, Promentis, Revance, Roche, Sanofi, Sunovion, Sun Pharma, Supernus, Teva, Theravance, and UCB. JL, RJ, and LL are former employees of Adamas Pharmaceuticals. AF is a former employee of Adamas Pharmaceuticals, and a current employee of Supernus Pharmaceuticals, Inc. Author CT reports research grants from Gateway LLC, Michael J Fox Foundation, Parkinson's Foundation, Parkinson's Study Group, and Roche/Genentech; grants and personal fees from Biogen Idee; and personal fees from 23andme, Acorda, Adamas Pharmaceuticals, Australia Parkinson's Mission, Cadent Therapeutics, CNS ratings, Gray Matter, Intec Pharma, Jazz Pharmaceuticals, Kyowa-Kirin Pharmaceuticals, Lundbeck Pharmaceuticals, Neurocrine Biosciences, Shake It Up Foundation, and Voyager Therapeutics. The authors declare that this study received funding from Adamas Pharmaceuticals, Inc and Supernus Pharmaceuticals, Inc. Adamas Pharmaceuticals, Inc had the following involvement in the study: Provided data and funding for MCIC calculations, medical writing support and participated in authorship of the paper. Supernus Pharmaceuticals had the following involvement in the study: Medical writing and editorial support.

## Publisher's note

All claims expressed in this article are solely those of the authors and do not necessarily represent those of their affiliated organizations, or those of the publisher, the editors and the reviewers. Any product that may be evaluated in this article, or claim that may be made by its manufacturer, is not guaranteed or endorsed by the publisher.

## Supplementary material

Supplementary Figure 1 Patient disposition.

Supplementary Table 1 Determination of minimal clinically important change^*^ in Unified Dyskinesia Rating Scale scores anchored to minimum measurable reductions in ON time with troublesome dyskinesia (as recorded in patient diaries) by treatment group and for overall patients.

Supplementary Table 2 Determination of minimal clinically important change^*^ in Unified Dyskinesia Rating Scale scores anchored to levels of improvement in the Clinical Global Impression of Change scale by treatment group and for overall patients.
