# Supplementary figures and images for: Clinically important change on the Unified Dyskinesia Rating Scale among patients with Parkinson's disease experiencing dyskinesia
Source: Front Neurol. 2022 Oct 20;13:846126. doi: 10.3389/fneur.2022.846126 (PMC9632663; doi:10.3389/fneur.2022.846126)

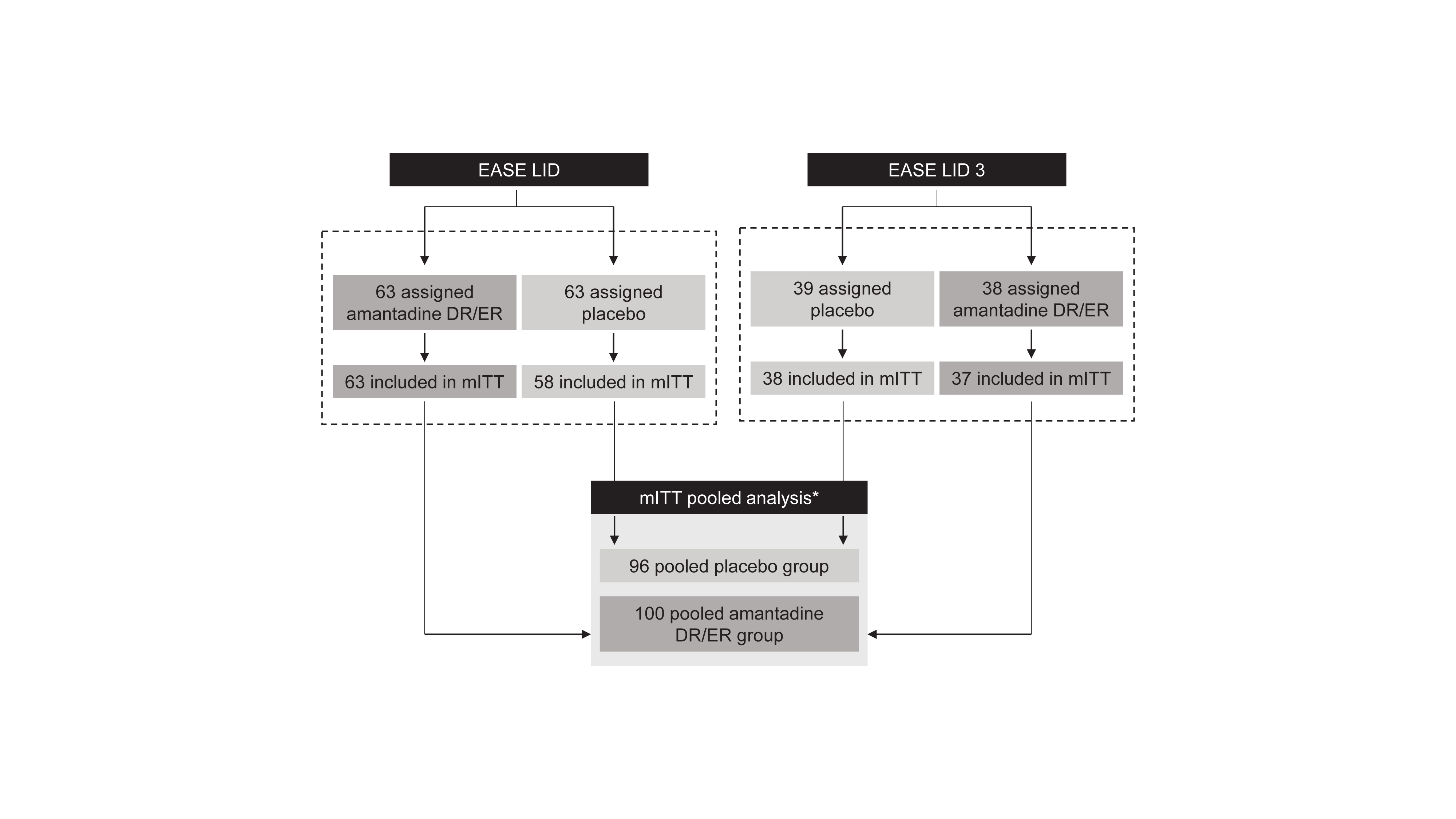

Supplement: Supplementary Figure 1 — Patient disposition. [file Image_1.jpg]
