# Supplementary material for: Clinically important change on the Unified Dyskinesia Rating Scale among patients with Parkinson's disease experiencing dyskinesia
Source: Front Neurol. 2022 Oct 20;13:846126. doi: 10.3389/fneur.2022.846126 (PMC9632663; doi:10.3389/fneur.2022.846126)
Supplement: Supplementary Table 1 — Determination of minimal clinically important change* in Unified Dyskinesia Rating Scale scores anchored to minimum measurable reductions in ON time with troublesome dyskinesia (as recorded in patient diaries) by treatment group and for overall patients. [file Table_1.DOCX]

**Supplementary Table 1.** Determination of minimal clinically important change* in Unified Dyskinesia Rating Scale scores anchored to significant time points for ON time with troublesome dyskinesia by treatment group

|  | **ON time with Troublesome Dyskinesia ≤−0.5 hour** | | | **ON time with Troublesome**  **Dyskinesia ≤−1 hour** | | |
| --- | --- | --- | --- | --- | --- | --- |
|  | Sensitivity | Specificity | MCIC | Sensitivity | Specificity | MCIC |
| **Total UDysRS** | |  |  |  |  |  |
| Placebo | 37/65 (56.9%) | 23/30 (76.7%) | −9 | 30/54 (55.6%) | 32/41 (88.0%) | −10 |
| Amantadine DR/ER | 69/82 (84.1%) | 11/13 (84.6%) | −8 | 62/77 (80.5%) | 14/18 (77.8%) | −9 |
| Total | 101/147 (68.7%) | 34/43 (79.1%) | −9^†^ | 90/13 (68.7%) | 46/59 (88.0%) | −10 |
| **Parts I + II** |  |  |  |  |  |  |
| Placebo | 42/65 (64.6%) | 17/30 (56.7%) | −2 | 34/54 (63.0%) | 25/41 (61.0%) | −3 |
| Amantadine DR/ER | 60/82 (73.2%) | 8/13 (61.5%) | −6 | 51/77 (66.2%) | 13/18 (72.2%) | −7 |
| Total | 99/147 (67.3%) | 27/43 (62.8%) | −5 | 85/13 (64.9%) | 39/59 (66.1%) | −6 |
| **Parts III + IV** | |  |  |  |  |  |
| Placebo | 37/65 (56.9%) | 22/30 (73.3%) | −3 | 30/54 (55.6%) | 26/41 (63.4%) | −3 |
| Amantadine DR/ER | 66/82 (80.5%) | 7/13 (53.8%) | −2 | 45/77 (58.4%) | 13/18 (72.2%) | −7 |
| Total | 98/147 (66.7%) | 29/43 (67.4%) | −3 | 89/131 (67.9%) | 36/59 (61.0%) | −3 |
| **Part I** |  |  |  |  |  |  |
| Placebo | 37/65 (56.9%) | 22/30 (73.3%) | −3.2 | 33/54 (61.1%) | 30/41 (73.2%) | −4 |
| Amantadine DR/ER | 52/82 (63.4%) | 11/13 (84.6%) | −6 | 50/77 (64.9%) | 14/18 (77.8%) | −6 |
| Total | 101/147 (68.7%) | 29/43 (67.4%) | −3.2 | 95/131 (72.5%) | 40/59 (67.8%) | −4 |
| **Part II** |  |  |  |  |  |  |
| Placebo | 22/65 (33.8%) | 22/30 (73.3%) | −2 | 19/54 (35.2%) | 30/41 (73.2%) | −2 |
| Amantadine DR/ER | 33/82 (40.2%) | 9/13 (69.2%) | −2 | 38/77 (49.4%) | 10/18 (55.6%) | −1 |
| Total | 55/147 (37.4%) | 31/43 (72.1%) | −2 | 50/131 (38.2%) | 42/59 (71.2%) | −2 |
| **Part III** |  |  |  |  |  |  |
| Placebo | 41/65 (63.1%) | 20/30 (66.7%) | −1 | 34/54 (63.0%) | 24/41 (58.5%) | −1 |
| Amantadine DR/ER | 47/82 (57.3%) | 8/13 (61.5%) | −4 | 46/77 (59.7%) | 12/18 (66.7%) | −4 |
| Total | 107/147 (72.8%) | 25/43 (58.1%) | −1 | 89/131 (67.9%) | 34/59 (57.6%) | −2 |
| **Part IV** |  |  |  |  |  |  |
| Placebo | 43/65 (66.2%) | 18/30 (60.0%) | −1 | 34/54 (63.0%) | 20/41 (48.8%) | −1 |
| Amantadine DR/ER | 54/82 (65.9%) | 9/13 (69.2%) | −2 | 52/77 (67.5%) | 12/18 (66.7%) | −2 |
| Total | 82/147 (55.8%) | 32/43 (74.4%) | −2 | 75/131 (57.3%) | 41/59 (69.5%) | −2 |

*The column labeled “MCIC” shows the threshold of UDysRS change that best predicted the decrease in ON time with troublesome dyskinesia shown in the column header. The MCIC for a decrease of at least 0.5 hours (the defined MCIC) is highlighted.

^†^Selected as the MCIC value for minimal improvement in UDysRS total score.

DR, delayed-release; ER, extended-release; MCIC, minimal clinically important change; UDysRS, Unified Dyskinesia Rating Scale.
