# Supplementary material for: Clinically important change on the Unified Dyskinesia Rating Scale among patients with Parkinson's disease experiencing dyskinesia
Source: Front Neurol. 2022 Oct 20;13:846126. doi: 10.3389/fneur.2022.846126 (PMC9632663; doi:10.3389/fneur.2022.846126)
Supplement: Supplementary Table 2 — Determination of minimal clinically important change* in Unified Dyskinesia Rating Scale scores anchored to levels of improvement in the Clinical Global Impression of Change scale by treatment group and for overall patients. [file Table_2.DOCX]

**Supplementary Table 2.** Determination of minimal clinically important change* in Unified Dyskinesia Rating Scale scores anchored to levels of improvement in the Clinical Global Impression of Change scale by treatment group

|  | | |  | | **CGI-C ≥0** | | | |  | | **CGI-C ≥1** | | |  | | **CGI-C ≥2** | | |  | **CGI-C ≥3** | | | | | |
| --- | --- | --- | --- | --- | --- | --- | --- | --- | --- | --- | --- | --- | --- | --- | --- | --- | --- | --- | --- | --- | --- | --- | --- | --- | --- |
|  |  | Sensitivity | | Specificity | | MCIC | |  | | Sensitivity | | Specificity | MCIC |  | Sensitivity | | Specificity | MCIC |  | | Sensitivity | | Specificity | | MCIC |
| **Total UDysRS** | | | | | | | | | | | | | | | | | | | | | | | | | |
| Placebo | | | 36/76 (47.4%) | | 12/20  (60.0%) | | −9 | |  | | 26/36  (72.2%) | 42/60 (70.0%) | −9 |  | | 11/15 (73.3%) | 55/81 (67.9%) | −11 |  | 2/2 (100.0%) | | 81/94 (86.2%) | | −21 | |
| Amantadine DR/ER | | | 63/93 (67.7%) | | 3/7  (42.9%) | | −10 | |  | | 44/76  (57.9%) | 17/24 (70.8%) | −17 |  | | 38/57 (66.7%) | 30/43 (69.8%) | −17 |  | 21/28 (75.0%) | | 49/72 (68.1%) | | −20 | |
| Total | | | 95/169 (56.2%) | | 16/27 (59.3%) | | −10 | |  | | 79/112 (70.5%) | 57/84 (67.9%) | −10^†^ |  | | 59/72 (81.9%) | 77/124 (62.1%) | −10 |  | 23/30 (76.7%) | | 129/166 (77.7%) | | −20 | |
| **Parts I + II** | | | | | | | | | | | | | | | | | | | | | | | | | |
| Placebo | | | 39/76 (51.3%) | | 9/20 (45.0%) | | −3 | |  | | 20/36 (55.6%) | 42/60 (70.0%) | −7 |  | | 11/15 (73.3%) | 42/81 (51.9%) | −3 |  | 2/2 (100.0%) | | 80/94 (85.1%) | | −15 | |
| Amantadine DR/ER | | | 37/93 (39.8%) | | 5/7  (71.4%) | | −12 | |  | | 34/76 (44.7%) | 19/24 (79.2%) | −12 |  | | 34/57 (59.6%) | 34/43  (79.1%) | −11 |  | 18/28 (64.3%) | | 56/72 (77.8%) | | −13 | |
| Total | | | 114/169 (67.5%) | | 10/27 (37.0%) | | −3 | |  | | 86/112 (76.8%) | 45/84 (53.6%) | −4 |  | | 55/72 (76.4%) | 71/124 (57.3%) | −6 |  | 20/30 (66.7%) | | 133/166 (80.1%) | | −13 | |
| **Parts III + IV** | | | | | | | | | | | | | | | | | | | | | | | | | |
| Placebo | | | 50/76  (65.8%) | | 8/20 (40.0%) | | 0 | |  | | 24/36 (66.7%) | 39/60 (65.0%) | −3 |  | | 11/15 (73.3%) | 58/81 (71.6%) | −5 |  | 2/2 (100.0%) | | 67/94 (71.3%) | | −6 | |
| Amantadine DR/ER | | | 58/93  (62.4%) | | 4/7  (57.1%) | | −5 | |  | | 33/76 (43.4%) | 18/24 (75.0%) | −9 |  | | 35/57 (61.4%) | 26/43 (60.5%) | −7 |  | 18/28 (64.3%) | | 51/72 (70.8%) | | −9 | |
| Total | | | 77/169 (45.6%) | | 19/27  (70.4%) | | −6 | |  | | 68/112 (60.7%) | 57/84 (67.9%) | −5 |  | | 51/72 (70.8%) | 80/124 (64.5%) | −5 |  | 19/30 (63.3%) | | 129/166 (77.7%) | | −9 | |
| **Part I** | | | | | | | | | | | | | | | | | | | | | | | | | |
| Placebo | | | 55/76 (72.4%) | | 7/20 (35.0%) | | 0 | |  | | 25/36 (69.4%) | 41/60 (68.3%) | −4 |  | | 13/15 (86.7%) | 50/81 (61.7%) | −4 |  | 2/2 (100.0%) | | 62/94 (66.0%) | | −7 | |
| Amantadine DR/ER | | | 61/93 (65.6%) | | 4/7  (57.1%) | | −5 | |  | | 48/76 (63.2%) | 16/24 (66.7%) | −6 |  | | 43/57 (75.4%) | 30/43 (69.8%) | −6 |  | 19/28 (67.9%) | | 56/72 (77.8%) | | −11 | |
| Total | | | 93/169 (55.0%) | | 15/27 (55.6%) | | −5 | |  | | 76/112 (67.9%) | 55/84 (65.5%) | −5 |  | | 52/72 (72.2%) | 84/124 (67.7%) | −6 |  | 20/30 (66.7%) | | 131/166 (78.9%) | | −11 | |
| **Part II** | | | | | | | | | | | | | | | | | | | | | | | | | |
| Placebo | | | 55/76 (72.4%) | | 6/20 (30.0%) | | 0 | |  | | 15/36 (41.7%) | 31/60 (51.7%) | −1 |  | | 9/15 (60.0%) | 21/81 (25.9%) | 0 |  | 1/2 (50.0%) | | 93/94 (98.9%) | | −10 | |
| Amantadine DR/ER | | | 80/93 (86.0%) | | 2/7  (28.6%) | | 1 | |  | | 31/76 (40.8%) | 16/24 (66.7%) | −2 |  | | 23/57 (40.4%) | 30/43 (69.8%) | −3 |  | 14/28 (50.0%) | | 47/72 (65.3%) | | −2 | |
| Total | | | 133/169 (78.7%) | | 8/27 (29.6%) | | 0 | |  | | 41/112 (36.6%) | 56/84 (66.7%) | −2 |  | | 25/72 (34.7%) | 90/124 (72.6%) | −3 |  | 15/30 (50.0%) | | 112/166 (67.5%) | | −2 | |
| **Part III** | | | | | | | | | | | | | | | | | | | | | | | | | |
| Placebo | | | 41/76 (53.9%) | | 10/20 (50.0%) | | −1 | |  | | 22/36 (61.1%) | 46/60 (76.7%) | −3 |  | | 11/15 (73.3%) | 56/81 (69.1%) | −3 |  | 2/2 (100.0%) | | 60/94 (63.8%) | | −3 | |
| Amantadine DR/ER | | | 61/93 (65.6%) | | 4/7  (57.1%) | | −3 | |  | | 37/76 (48.7%) | 14/24 (58.3%) | −5 |  | | 36/57 (63.2%) | 25/43 (58.1%) | −4 |  | 17/28 (60.7%) | | 49/72 (68.1%) | | −6 | |
| Total | | | 90/169 (53.3%) | | 17/27 (63.0%) | | −3 | |  | | 72/112 (64.3%) | 56/84 (66.7%) | −3 |  | | 53/72 (73.6%) | 77/124 (62.1%) | −3 |  | 22/30 (73.3%) | | 103/166 (62.0%) | | −4 | |
| **Part IV** | | | | | | | | | | | | | | | | | | | | | | | | | |
| Placebo | | | 46/76 (60.5%) | | 11/20  (55.0%) | | −1 | |  | | 17/36 (47.2%) | 42/60 (70.0%) | −2 |  | | 11/15  (73.3%) | 57/81 (70.4%) | −2 |  | 2/2 (100.0%) | | 73/94 (77.7%) | | −3 | |
| Amantadine DR/ER | | | 72/93 (77.4%) | | 3/7  (42.9%) | | −1 | |  | | 49/76 (64.5%) | 12/24 (50.0%) | −2 |  | | 43/57 (75.4%) | 25/43 (58.1%) | −2 |  | 20/28 (71.4%) | | 44/72 (61.1%) | | −3 | |
| Total | | | 118/169 (69.8%) | | 14/27 (51.9%) | | −1 | |  | | 66/112 (58.9%) | 54/84 (64.3%) | −2 |  | | 54/72 (75.0%) | 82/124 (66.1%) | −2 |  | 22/30 (73.3%) | | 117/166 (70.5%) | | −3 | |

*The column labeled “MCIC” shows the threshold of UDysRS change that best predicted the level of CGI-C improvement shown in the column header. The MCIC for at least minimal improvement in the CGI-C (the defined MCIC) is highlighted.

^†^Selected as the MCIC value for minimal improvement in UDysRS total score.

CGI-C, Clinical Global Impression of Change; DR, delayed-release; ER, extended-release; MCIC, minimal clinically important change; UDysRS, Unified Dyskinesia Rating Scale.
